# Glycerol-3-Phosphate Acyltransferase-2 Is Expressed in Spermatic Germ Cells and Incorporates Arachidonic Acid into Triacylglycerols

**DOI:** 10.1371/journal.pone.0042986

**Published:** 2012-08-08

**Authors:** Elizabeth R. Cattaneo, Magali Pellon-Maison, Martin E. Rabassa, Ezequiel Lacunza, Rosalind A. Coleman, Maria R. Gonzalez-Baro

**Affiliations:** 1 Instituto de Investigaciones Bioquímicas de La Plata, Consejo Nacional de Investigaciones Cientificas y Tecnicas – Facultad de Ciencias Medicas, Universidad Nacional de La Plata, La Plata, Argentina; 2 Centro de Investigaciones Inmunológicas Básicas y Aplicadas, Facultad de Ciencias Médicas, Universidad Nacional de La Plata, La Plata, Argentina; 3 Department of Nutrition, University of North Carolina, Chapel Hill, North Carolina, United States of America; University of Delhi, India

## Abstract

**Background:**

De novo glycerolipid synthesis begins with the acylation of glycerol-3 phosphate catalyzed by glycerol-3-phosphate acyltransferase (GPAT). In mammals, at least four GPAT isoforms have been described, differing in their cell and tissue locations and sensitivity to sulfhydryl reagents. In this work we show that mitochondrial GPAT2 overexpression in CHO-K1 cells increased TAG content and both GPAT and AGPAT activities 2-fold with arachidonoyl-CoA as a substrate, indicating specificity for this fatty acid.

**Methods and Results:**

Incubation of GPAT2-transfected CHO-K1 cells with [1-^14^C]arachidonate for 3 h increased incorporation of [^14^C]arachidonate into TAG by 40%. Consistently, arachidonic acid was present in the TAG fraction of cells that overexpressed GPAT2, but not in control cells, corroborating GPAT2's role in synthesizing TAG that is rich in arachidonic acid. In rat and mouse testis, *Gpat2* mRNA was expressed only in primary spermatocytes; the protein was also detected in late stages of spermatogenesis. During rat sexual maturation, both the testicular TAG content and the arachidonic acid content in the TAG fraction peaked at 30 d, matching the highest expression of *Gpat2* mRNA and protein.

**Conclusions:**

These results strongly suggest that GPAT2 expression is linked to arachidonoyl-CoA incorporation into TAG in spermatogenic germ cells.

## Introduction

The de novo synthesis of glycerolipids begins with the acylation of glycerol-3-phosphate which is catalyzed by glycerol-3-phosphate acyltransferase (GPAT, EC 2.3.1.15). Four different genes encode GPAT isoforms 1–4, which differ in tissue expression pattern, subcellular location, fatty acyl-CoA substrate preference, and sensitivity to *N*-ethylmaleimide (NEM) [1]. The GPAT product, lysophosphatidic acid (LPA) is subsequently acylated to phosphatidic acid (PA) by 1-acylglycerol-3-phosphate acyltransferase (AGPAT), which also consists of several isoforms. PA is the precursor for the synthesis of both triacylglycerol (TAG) and glycerophospholipids. Both the specific activity and regulation of the different GPAT isoforms differ, depending on the tissue and the metabolic state of the animal [2], [3].

In this work, we focus on the function of GPAT2, a mitochondrial isoform that was first identified in liver mitochondria from *Gpat1−/−* mice. Characterization of GPAT2 activity in liver mitochondria from *Gpat1−/−* mice showed that, unlike GPAT1, GPAT2 is inactivated by NEM, and has no preference for palmitoyl-CoA compared with oleoyl-CoA [4]. Even though GPAT2 heterologous overexpression leads to enhanced TAG synthesis, the abundance of the mRNA transcript of *Gpat2* is 50-fold higher in testis than in lipogenic tissues, and *Gpat2* mRNA abundance is not altered by fasting or refeeding [5], as is observed for *Gpat1*, suggesting that GPAT2 might synthesize TAG that would not be used as an energy store [5].

The most striking feature of testicular lipids is the fatty acid composition of both neutral and polar lipids. Testicular lipids are unusually rich in long-chain polyunsaturated fatty acids of 18 to 22 carbons and very-long-chain polyunsaturated fatty acids of 24 to 32 carbons. Long chain fatty acids are required for the synthesis of spermatogenic cell membranes, and are essential for the acrosomal reaction and fertilization [6], [7]. Very-long-chain fatty acids are required in the spermatozoon for the physiological events that precede fertilization. Because GPAT2 is abundant in testis, we hypothesized that this acyltransferase might contribute to the metabolism of polyunsaturated fatty acids. In this work, we show that GPAT2 specifically incorporates arachidonic acid (AA) to TAG.

## Materials and Methods

### Ethics Statement

All the studies performed with rats were approved by the Directive Board of the INIBIOLP and were carried out in accordance with the AVMA Animal Welfare Policies (http://www.avma.org/issues/animal_welfare/policies.asp) and AVMA Guidelines on Euthanasia (http://www.avma.org/issues/animal_welfare/euthanasia.pdf). (Instituto de Investigaciones Bioquimicas de La Plata's Animal Welfare Assurance No. A5647–01).

### Subcellular fractions of rat testis

Nineteen, 30, 40 and 60 d-old male Wistar rats were killed by decapitation. Testes were removed, rinsed with ice cold PBS and submerged in precooled buffer H (10 mM Hepes-KOH, pH 7.4, 0.25 M sucrose, 1 mM EDTA and 1 mM dithiothreitol) with 0.002% v/v protease inhibitor cocktail (general use, Sigma) 1∶6 (w/v). The total cellular homogenate was obtained by homogenizing with 10 up-and-down strokes in a Teflon-glass vessel. Large debris and nuclei were pelleted by centrifuging twice at 600×g for 5 min. The supernatant (post-nuclear homogenate) was centrifuged for 10 min at 10,000×g. The pellet was resuspended in 1 ml of buffer H/g of testis and homogenized in a Dounce tissue grinder with a glass pestle to obtain the crude mitochondria fraction. The supernatant was centrifuged at 110,000×g for 60 min to obtain the microsomal fraction. Mitochondria were further purified by a self-forming Percoll gradient centrifugation, as described [8]. Briefly, 1 ml of crude mitochondria suspension was loaded in a tube containing 9 ml 30% (v/v) Percoll in buffer H and centrifuged at 95,000×g for 30 min in a 70.1 Ti rotor, in a Beckman LE-80K ultracentrifuge. After centrifugation, the tubes contained two distinct bands separated by a clear zone. The lowest brownish bands were collected, pooled and diluted 4-fold in buffer H. To remove the Percoll, the samples were centrifuged at 6,300×g for 10 min and washed twice with buffer H; the resulting fraction was highly enriched in mitochondria. The upper bands contained the mitochondrial-associated membrane fractions. The NEM-sensitive GPAT activity present in this fraction corresponded to the microsomal isoforms [8]. The purity of the mitochondria was confirmed by assaying the ER marker enzyme NADPH-cytochrome c reductase and by Western blot with the mitochondrial marker protein VDAC (porin).

### Enzyme assays

GPAT activity was assayed as described previously [9]. Assays contained 0.8 mM glycerol-3-phosphate (2 µCi [^3^H] glycerol-3-phosphate, [4]), 75 mM Tris-HCl pH 7.4, 4 mM MgCl_2_, 2 mg/ml BSA, 8 mM NaF, 1 mM dithiothreitol and 60 µM of the corresponding acyl-CoA, and were incubated at 37°C for 10 min. Radioactivity in chloroform-soluble reaction products was quantified by liquid scintillation counting after several washes with 1% perchloric acid. NEM-sensitive GPAT activity was calculated by subtracting the NEM-resistant activity from the total. AGPAT activity was assayed as described previously [10], using 10 µM [^3^H]oleoyl-lysophosphatidic acid (Perkin Elmer) and 60 µM oleoyl-CoA or arachidonoyl-CoA (Sigma) as substrates at 37°C for 7 min. The reaction was stopped with 1% perchloric acid, the radioactive lipids extracted and the solvent evaporated. Lipids were spotted on a Silica G 60 plate (Merck) together with standards for LPA, PA and diacylglycerol. Chromatograms were developed in CHCl_3_/pyridine/formic acid (88%) (50∶30∶7). Standards were visualized by iodine vapours, the spots corresponding to LPA and PA were scrapped and the radioactivity quantified by liquid scintillation counting. Cytochrome c NADPH oxidoreductase (ER marker) was measured using a commercial kit (Sigma). To determine the products of the GPAT reaction, products from the GPAT assay using total membranes of control, GPAT1- and GPAT2-overexpressing CHO-K1 cells and [U^14^C]-glycerol-3-phosphate (Perkin-Elmer) as substrate were pooled, dried and resuspended in chloroform. LPA and PA (10 µg/sample) were added as carrier lipids for TLC analysis as described above. Standards were visualized under iodine vapor, and the radioactivity associated with each spot was quantified in a Storm 840 scanner (GE Healthcare).

### Immunoblotting

**Figure 1:**
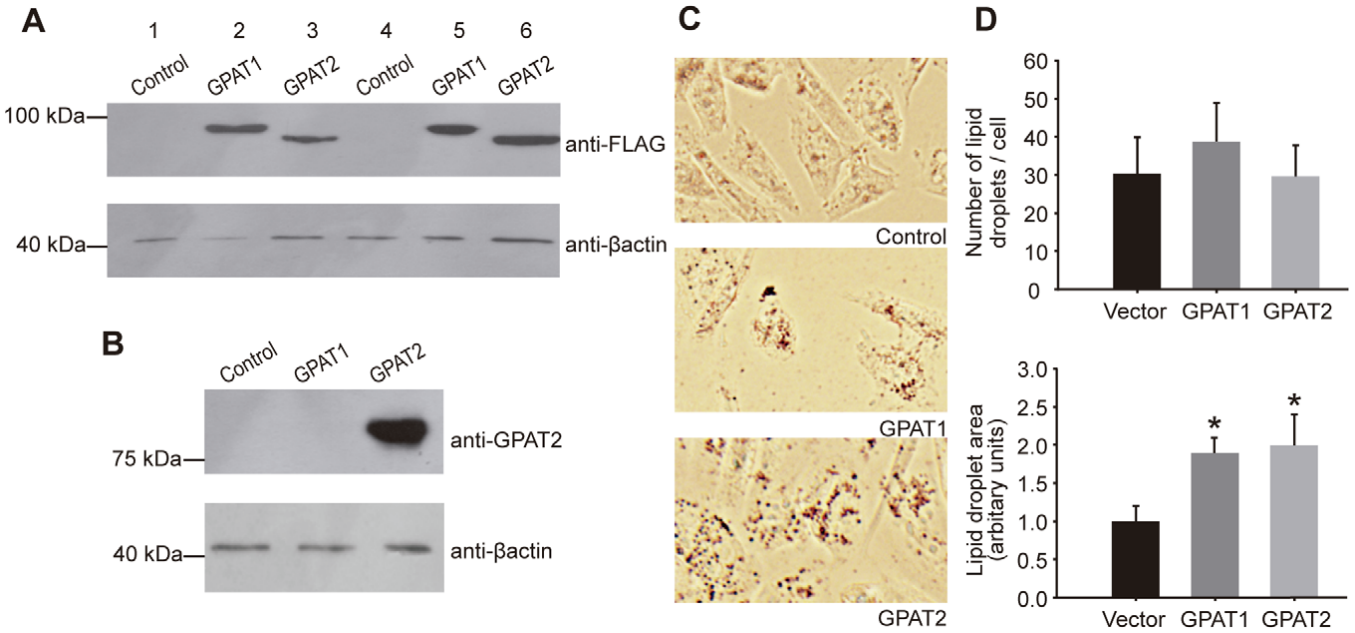
GPAT2 overexpression increased TAG storage in CHO-K1 cells. CHO-K1 cells were transiently transfected with pcDNA3.1 empty vector (control), pcDNA3.1-GPAT1 (GPAT1) or pcDNA3.1-GPAT2 (GPAT2) constructs tagged with a FLAG epitope (Lanes 1–3 and 4–6 correspond to two different transient transfections). The expression of GPAT1 and GPAT2 was confirmed by western blot. Total particulate protein (50 µg) from GPAT1, GPAT2 and control cells was probed with anti-FLAG (A) and anti-GPAT2 (B) antibodies. The molecular mass of the expressed protein was 90 kDa (GPAT1) and 80 kDa (GPAT2). The membranes were probed with anti-β-actin antibody as a loading control. C) Lipid droplets were visualized in control, GPAT1, and GPAT2-overexpressing CHO-K1 cells by Oil-Red O staining. D) The average size of cellular lipid droplets and the average number of lipid droplets in each cell were quantified by Image Pro plus v5.1 software. Data represent mean ± SD of three independent experiments. (*p<0.05).

One-hundred µg of total proteins of mitochondria-enriched fraction isolated from rat testis were separated on 8 or 12% SDS-PAGE, transferred to a polyvinylidene difluoride membrane (Bio-Rad) and probed with 1/1000 anti-voltage dependent anion channel (anti-VDAC, Affinity Bioreagents). Total membranes (50 µg protein) from cells overexpressing FLAG-tagged recombinant GPAT2 were separated in 8% SDS-PAGE and probed with 1∶3300 of M2 anti-FLAG antibody (Sigma) as previously described [9]. Anti-β-actin antibody (Abcam ab8227) at a dilution 1∶2500 was used as a gel-loading control. Endogenous GPAT2 was probed in 100 µg of rat testis cellular fractions with an anti-human GPAT2 antibody (Sigma HPA036841), which also recognizes mouse and rat GPAT2 (Fig. 1). Membranes were then washed extensively and probed with horseradish peroxidase-conjugated goat anti-mouse or anti-rabbit IgG antibody (Thermo-Pierce). For chemiluminescent detection, the membranes were incubated with Super Signal detection kit (Thermo-Pierce).

### Transient transfection and analysis of epitope-tagged GPAT2 in CHO-K1 and Vero cells

CHO-K1 and Vero cells were routinely grown in MEM and DMEM, respectively, containing 10% fetal bovine serum, 100 U/ml penicillin, and 100 µg/ml streptomycin at 37°C with 5% CO_2_. Cells were grown in 60-mm dishes to 90% confluence and then transfected with 5 µg of the cDNA encoding the complete open reading frame of either mouse GPAT2 or rat GPAT1 (tagged with a FLAG epitope at the C-terminus) cloned in pcDNA3.1 vector (BamHI- XhoI sites) or with the empty vector as a control. The cloning, identification and 3'tagging of GPAT2's cDNA have been previously reported [5]. GPAT1 and GPAT2 cDNA sequence was confirmed at the UNC Genome Analysis Core Facility. Cationic liposomes (Lipofectamine 2000, Invitrogen) were used according to the manufacturer's instructions. GPAT2 expression was monitored by western blot.

To obtain a total membrane preparation, 24 h after transfection, the cells were rinsed with PBS, scraped into buffer H containing a protease inhibitor cocktail (Sigma) and homogenized with 10 up-and-down strokes in a Teflon-glass homogenizing vessel. After centrifugation at 16,000×g for 15 min in a microcentrifuge at 4°C, the total particulate pellet (membrane fraction) was resuspended in buffer H, separated into aliquots, and stored at −70°C. Protein was determined with bovine serum albumin as standard [11]. This fraction was used to measure GPAT activities.

To assess the TAG content of GPAT2-overexpressing CHO-K1 cells, lipid droplets were stained with Oil Red O. Twenty-four hours after transfection, the cells were rinsed with PBS, fixed with 10% formaldehyde for 15 min at room temperature and then incubated with 3 mg/ml of Oil Red O stain (Sigma, prepared from a stock solution 0.5 g of Oil Red O in 100 ml of isopropanol) for 1 h at room temperature. Plates were visualized in an Olympus BX51 microscope and analyzed by Image PRO v 5.1 software (Media Cybernetics).

### Cell radiolabeling, lipid and fatty acid analysis

CHO-K1 cells were seeded in 6-well plates and transfected with pcDNA3.1-GPAT2-FLAG or pcDNA3.1 (control) for 18 h, then incubated with trace (4.3 µM) [^14^C]AA (Perkin Elmer, 58.2 µCi/µmol, 0.25 µCi/well) in a final volume of 1 ml of routine medium supplemented with 0.5% BSA. After three hours, the medium was removed, and the cells were washed with 0.1% BSA in ice-cold PBS scraped in ice-cold methanol and H_2_O. Total lipids were extracted [12] and separated by TLC on silica-gel 60 plates (Merck), with a mobile phase composed of hexane-ethyl ether-acetic acid (80∶20∶1; v/v) for neutral lipids and chloroform∶ methanol∶ acetic acid∶ water (50∶37.5∶3.5∶1.5; v/v) for phospholipid species. All samples were chromatographed in parallel with pure lipid standards. The ^14^C-labeled lipids were detected using a Bioscan Image System.

To analyze the fatty acids from TAG fraction of GPAT2 and control CHO-K1 cells, 24 h after transfection lipids were extracted and separated by TLC as described above. TAG was scraped from the plate and eluted with hexane∶chloroform∶methanol (3∶2∶1, v/v). Fatty acid methyl esters obtained by reacting with BF_3_ in methanol were analyzed by gas liquid chromatography in a Hewlett-Packard HP 6890 chromatograph equipped with an Omega Wax capillary column [13].

### mRNA quantification

*Northern blot:* Total RNA from testis of 19, 30, 40 and 60 d-old rats was isolated with TRIzol Reagent (Invitrogen) according to the manufacturer's instructions. Twenty µg of total RNA were size-fractionated on a 1% agarose-formaldehyde gel and then transferred to a Hybond-XL nylon membrane (GE- Healthcare). The *Gpat2* and β-actin probes were prepared by incorporating [^32^P]dCTP by random prime labeling (Prime-a-Gene**®** Labeling System, Promega). The cDNA template for the *Gpat2* probe was PCR amplified using the forward primer CAGCATCTGAGTGCAAAGC and the reverse primer ACAGGTGGAGCTGGGGTCCTG, which perfectly match the mouse and rat *Gpat2* ORFs. The amplification product corresponded to nucleotides 1951–2335. Northern blot hybridization was performed as described by Sambrook et al. [14]. The radioactive signals were quantified using a Phosphorimager scanner (Molecular Dynamics) and 1D Image Analysis Software (Kodak).

*Real-time quantitative PCR* analysis was performed using an MX3000 apparatus (Statagene). Total RNA was isolated with the total RNA minikit (Bio-Rad). Total RNA (1 µg) from individual rats was used to generate cDNA using the iScript cDNA synthesis kit (Bio-Rad). Equal amounts of cDNA (derived from 200 ng of total RNA) were amplified in triplicate with IQ Sybr Green Super Mix (Bio-Rad). Primers were designed to amplify the region between 1789–1975 of the open reading frame from both rat and mouse *Gpat2* (forward primer: ATCCTACTGCTGCTGCACCT included in exon 15 and reverse ACAGCAGCTTTGCACTCAGA included in exon 17). The thermal profile was 50°C for 10 min, 95°C for 5 min, and 40 cycles of 95°C for 30 s, 60°C for 1 min and 72°C for 30 s. The products were quantified by the standard curve method.

### TAG quantification and fatty acid composition from rat testis

Testes from 19, 30, 40 and 60 d-old rats were dissected and total lipids were extracted. Neutral lipids were separated by TLC as described above. TAG was quantified by scanning the plate in a STORM 840 scanner (GE Healthcare) after charring with 5% sulfuric acid in methanol at 180°C for 15 min. Alternatively, TAG was scraped from the plate and fatty acid composition was determined by GC as described above.

### In situ mRNA hybridization

Constructs for synthesizing the riboprobes for in situ hybridization were prepared by digesting *Gpat2* cDNA from the pcDNA3.1-*Gpat2* construct in either the BamHI and EcoRI sites and subcloning the 1456 bp fragment into pGEM11z(f)+ vector (Promega). The tissue preparation, probe synthesis, and hybridization were performed by the In Situ Hybridization Core Facility at the University of North Carolina, Chapel Hill (http://www.med.unc.edu/neuroscience/core-facilities/in-situ-hybridization). Adult mouse testes were fixed in 4% paraformaldehyde in 0.1 M PBS, embedded in a freezing medium (O.C.T. compound, Tissue-Tek) and cut in 10 µm sections. One µg of linearized and purified *Gpat*2 construct was used to synthesize the antisense probe using T7-RNA polymerase and the sense probe using SP6-RNA polymerase in the presence of digoxigenin-UTP (Roche Applied Science). After proteinase-K treatment and prehybridization, digoxigenin-labeled riboprobes were hybridized for 16 h at 65°C in hybridization oven. After rinsing, sections were incubated with a 1∶2000 dilution of alkaline phosphatase conjugated sheep anti-digoxigenin antibody (Roche Diagnostics) in blocking buffer at room temperature for 3 h and developed in a nitroblue tetrazolium/5-bromo-4-chloro-3-indolyl phosphate solution (20 µl/ml; Roche) in the dark for 16–20 h. After developing, material was rinsed, and alkaline phosphatase activity was quenched by fixation in 4% paraformaldehyde; sections were mounted in aqueous mounting medium (Faramount aqueous mounting medium, DAKO). Slides were visualized in an Olympus BX52 microscope.

### Immunofluorescence and Immunohistochemistry analysis

Adult and juvenile rat testes were fixed in Bouin's solution and sections 4 µm thick were cut from block paraffin tissue samples and then deparaffinized. For immunofluorescence, sections were blocked with 10% normal horse serum in 1% bovine serum albumin (Sigma) in PBS, slides were incubated with rabbit antihuman polyclonal antibody against GPAT2 (1∶100) or PBS (negative controls) overnight at 4°C and secondary Alexa-Fluor® 488-labeled anti-rabbit immunoglobulin (1∶150; Thermo-Pierce). Nuclei were stained with propidium iodide (Invitrogen). Slides were visualized in an Olympus BX52 fluorescence microscope. For immunohistochemistry analysis, sections were incubated in 1% H_2_O_2_ in methanol for 30 min, washed with PBS and then incubated with 10% normal horse serum in 1% bovine serum albumin (Sigma) in PBS. Slides were sequentially incubated for 10 min in 10 mM citrate buffer, pH 6, at 100°C and washed with PBS and with rabbit anti-human polyclonal antibody against GPAT2 (1∶35) or PBS (negative controls) overnight at 4°C and secondary HRP-conjugated anti-rabbit immunoglobulin (1∶150; Thermo-Pierce). The reaction was developed with the LSAB2/HRP kit and liquid 3,3′-diaminobenzidine (Dako) according to the manufacturer's recommendations. Slides were counter-stained with haematoxylin to visualize the nuclei and analyzed in an Olympus BX52 microscope.

### Statistics

Differences between the control and transfected cells were analyzed by Student's t-test. Results were considered significant at the 5% level.

## Results

### GPAT2 overexpression in CHO-K1 cells increased arachidonoyl-CoA esterification and TAG accumulation

Because i) heterologous GPAT2 expression increased exogenous fatty acid incorporation into TAG [5], and ii) CHO-K1 cells do not export TAG to the media, we hypothesized that GPAT2 overexpression in CHO-K1 cells would increase TAG stores in these cells. To test our hypothesis, we transiently transfected FLAG-tagged *Gpat2* inserted into pcDNA3.1 vector. The empty vector was used as a negative control, and FLAG-tagged GPAT1 [15] was used as a control for transfection. Both proteins showed similar levels of expression (Fig. 1A). The anti-GPAT2 antibody (Sigma HPA036841) did not cross-react with the other mitochondrial isoform, GPAT1 (Fig. 1B), and CHO-K1 cells do not express GPAT2 protein (the absence of *Gpat2* mRNA in CHO-K1 cells was corroborated by qPCR). To establish the role of GPAT2 in cellular TAG storage, lipid droplets of GPAT1-, GPAT2-overexpressing and control cells were visualized by Oil-Red O staining (Fig. 1C). The average numbers of lipid droplets in each cell and the average size of cellular lipid droplets were quantified (Fig. 1D). Consistent with its role in TAG synthesis [15], GPAT1 overexpression increased the size of cellular lipid droplets. Similarly, with GPAT2 overexpression, the droplet number remained unchanged, but the lipid droplet size doubled.

**Figure 2:**
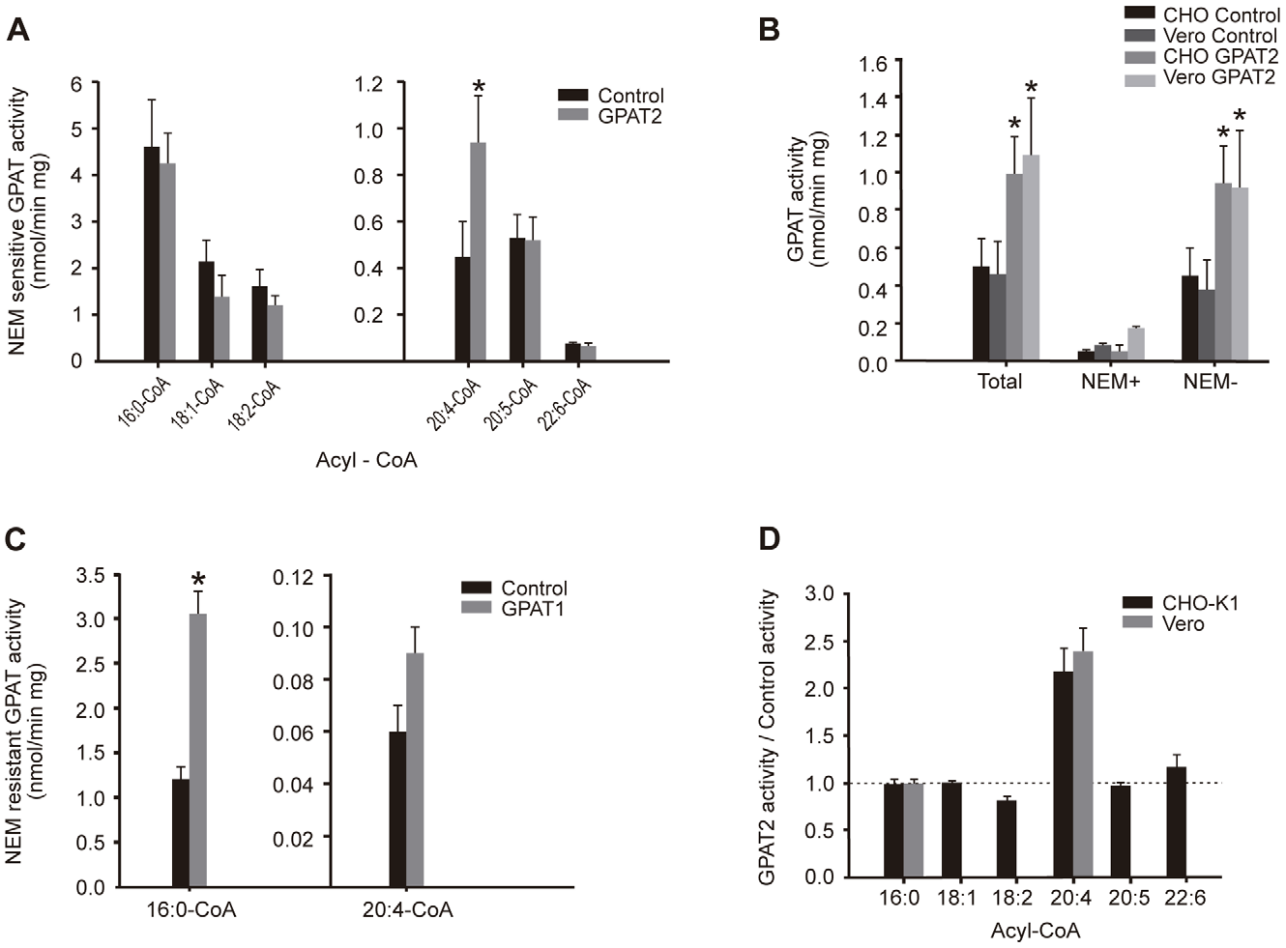
GPAT2 overexpression increased arachidonoyl-CoA esterification. CHO-K1 cells were transiently transfected with pcDNA3.1 empty vector (control), pcDNA3.1-GPAT1 (GPAT1) or pcDNA3.1-GPAT2 (GPAT2) constructs. A) NEM-sensitive GPAT activity was measured with [^3^H]glycerol-3-phosphate and the acyl-CoA esters substrates palmitoyl-CoA (16∶0-CoA), oleoyl-CoA (18∶1-CoA), linoleoyl-CoA (18∶2-CoA), arachidonoyl-CoA (20∶4-CoA), eicosapentaenoyl-CoA (20∶5-CoA) and docosahexanoyl-CoA (22∶6-CoA) in CHO-K1 cells. GPAT2 overexpression significantly increased GPAT activity only when arachidonoyl-CoA was used as a substrate. B) GPAT activity was measured in both control and GPAT2-overexpressing CHO-K1 and Vero cells with [^3^H]glycerol-3-phosphate and arachidonoyl-CoA in the absence (Total GPAT activity) and presence (NEM-resistant, NEM+) of 2 mM NEM. NEM-sensitive GPAT activity (NEM−) was calculated by difference of the other two activity values. C) NEM-resistant GPAT activity was measured in control and GPAT1-overexpressing CHO-K1 cells with the substrates palmitoyl-CoA (16∶0-CoA) and arachidonoyl-CoA (20∶4-CoA). D) The ratio between NEM-sensitive GPAT activity in control and GPAT2-overexpressing CHO-K1 cells with the same fatty acyl-CoA substrates as in A) and Vero cells with palmitoyl-CoA and arachidonoyl-CoA was calculated. Bars represent the mean ± SD of three independent experiments (**p<0.01).

To determine the substrate specificity for GPAT2, glycerol-3-phosphate acyltransferase activity was assayed in total membranes obtained from vector-transfected CHO-K1 control cells and cells transiently overexpressing GPAT2. No statistically significant differences were found in either total or NEM-sensitive GPAT activity in control or GPAT2-overexpressing cells when palmitoyl-CoA, oleoyl-CoA, or linoleoyl-CoA was used as substrate (Fig. 2A). However, total GPAT specific activity was 2-fold higher in GPAT2-overexpressing cells when arachidonoyl-CoA was used, and this activity was sensitive to NEM inactivation (Fig. 2B). This result was corroborated overexpressing GPAT2 in the Vero cell line (Fig. 2B). NEM-resistant GPAT activity was unchanged by GPAT2 expression with all substrates assayed (results not shown). CHO-K1 cells that overexpressed GPAT1 were used as a positive control for transfection and GPAT activity assays, and consistently showed a ∼3-fold increase in NEM resistant GPAT activity with palmitoyl-CoA, whereas the change in activity with arachidonoyl-CoA was not significant (Fig. 2C). To confirm that the increase in activity with arachidonoyl-CoA was specific for this polyunsaturated acyl-CoA, we measured GPAT activity with docosahexanoyl-CoA (22∶6 n-3) and eicosapentaenoyl-CoA (20∶5 n-3). Neither of these substrates was used by GPAT2 (Fig. 2A). Activity results expressed as percentage of control are summarized in Fig. 2D.

**Figure 3:**
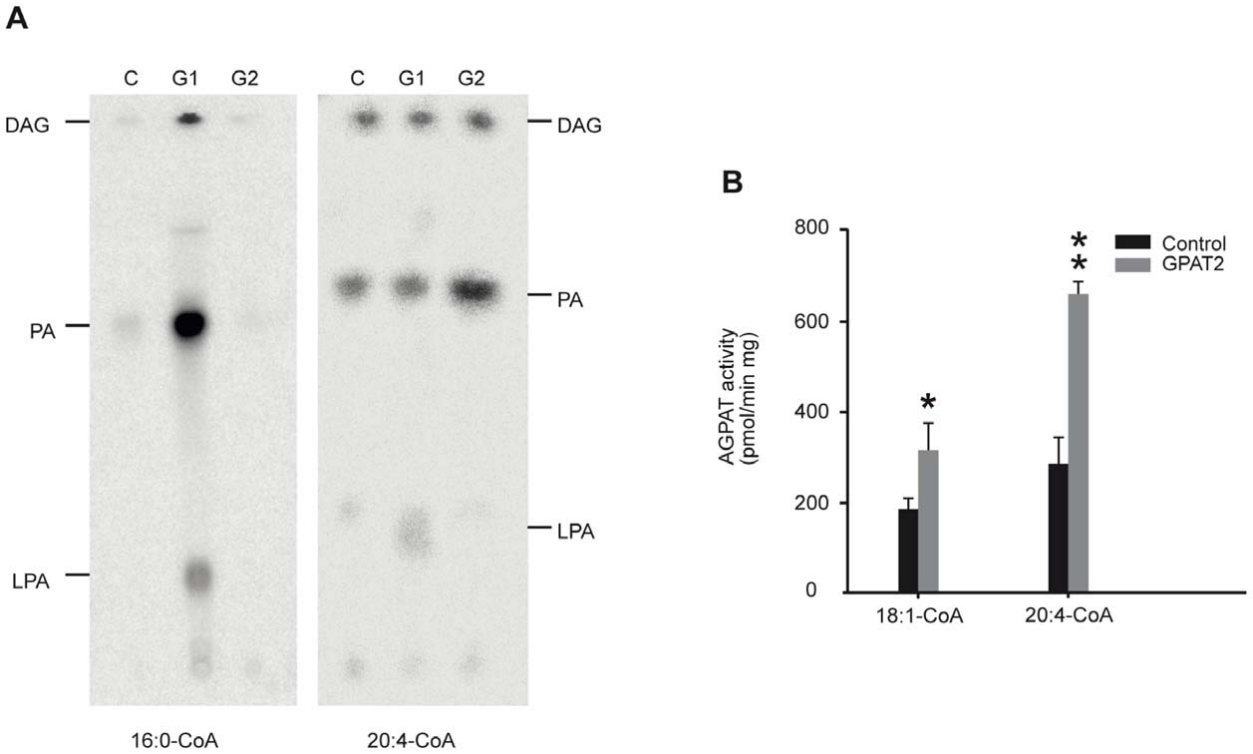
GPAT2 overexpression increased phosphatidic acid synthesis. A) The reaction products of GPAT reaction measured in control (C), GPAT1 (G1) and GPAT2 (G2)-overexpressing CHO-K1 cells with [^14^C]glycerol-3-phosphate and palmitoyl-CoA (16∶0-CoA) or arachidonoyl-CoA (20∶4-CoA) were visualized by a Storm radioactivity scanner. DAG, diacylglycerol, PA, phosphatidic acid, LPA, lysophosphatidic acid. B) AGPAT activity was measured with oleoyl-CoA or arachidonoyl-CoA and [^14^C]oleoyl-lysophosphatidic acid. GPAT2 overexpression significantly increased both GPAT and AGPAT activities only when arachidonoyl-CoA was used as a substrate (*p<0.05, **p<0.01).

We analyzed the products of GPAT reaction in CHO-K1 control, GPAT1- and GPAT2-overexpressing cells when both arachidonoyl-CoA and palmitoyl-CoA were used as substrates (Fig. 3A). With arachidonoyl-CoA, GPAT2 overexpression increased the synthesis of PA, whereas the synthesis of LPA was not affected. In GPAT1 cells incubated with arachidonoyl-CoA, a small increase in incorporation into LPA was observed, but no differences were found in PA compared to control cells. In contrast, only GPAT1 overexpression increased the incorporation of palmitoyl-CoA into LPA, PA and diacylglycerols, with PA as the major product. With palmitoyl-CoA, no differences were observed in the products of GPAT2 and control cells.

GPAT enzymes catalyze the limiting step in PA synthesis, so most of the LPA synthesized by the GPAT reaction is immediately acylated via reactions catalyzed by AGPAT proteins to yield PA [16]. Because we could not detect any LPA product on the TLC plates when GPAT2 was overexpressed, we compared AGPAT activity in total membranes isolated from CHO-K1 cells that did or did not (control) overexpress GPAT2 (Fig. 3B). GPAT2 overexpression increased AGPAT activity ∼50% with oleoyl-CoA and 2- fold when arachidonoyl-CoA was used as substrate, confirming the preference of GPAT2 for this fatty acid.

### GPAT2 overexpression enhances the incorporation of AA into TAG

**Figure 4:**
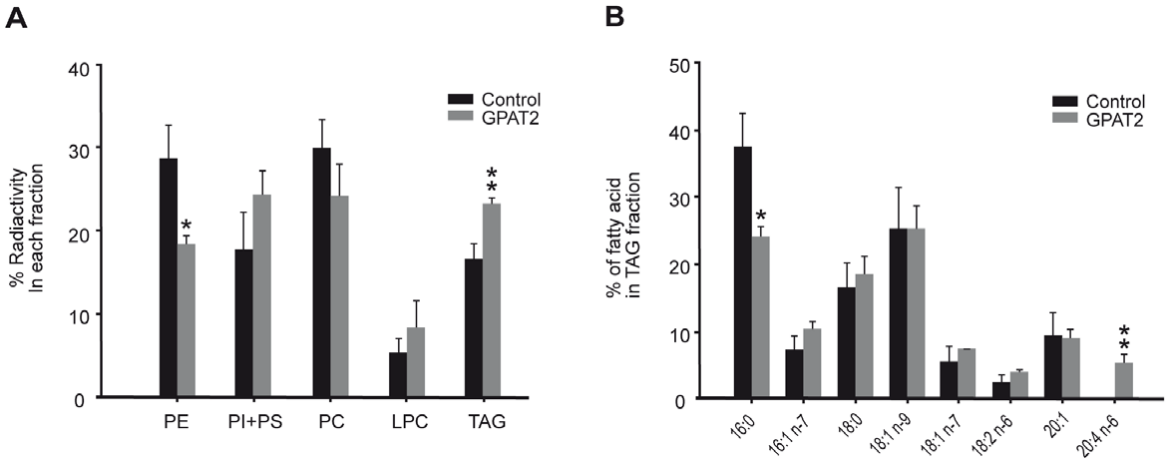
GPAT2 overexpression stimulated AA incorporation in TAG. CHO-K1 cells were transiently transfected with pcDNA3.1 empty vector (control) or pcDNA3.1-GPAT2 (GPAT2) constructs. A) Twenty-four h after transfection, cells growing in 6-well plates were incubated with 0.25 µCi of [^14^C]AA/well in standard medium plus 0.5% BSA for 3 h. Lipids were extracted and separated as described under “Materials and Methods”. Bars represent the mean ± SD of three independent experiments; PE, phosphatidylethanolamine, PI, phosphatidylinositol, PS, phosphatidylserine, PC, phosphatidylcholine, LPC, lysophosphatidylcholine, TAG, triacylglycerol. B) Twenty-four h after transfection, total lipids were extracted from control and GPAT2-transfected cells incubated with medium plus 10% FBS, and separated by TLC to isolate the TAG fraction and analyze the fatty acid composition. Values represent the mean ± SD of three independent experiments (*p<0.05, **p<0.01).

Because arachidonoyl-CoA appeared to be the preferred substrate for GPAT2, we measured the incorporation of [1-^14^C]AA into complex lipids in cells that transiently overexpressed GPAT2 (Fig. 4A). Compared to control cells after a 3-h incubation, GPAT2 cells showed a 40% higher incorporation of [1-^14^C]AA into TAG, as well as diminished incorporation of [1-^14^C]AA into phosphatidylethanolamine (Fig. 4A). This result was surprising because AA, like other polyunsaturated fatty acids, is primarily found in the phospholipid fraction of CHO-K1 cells [17]. Because transient transfection of GPAT2 also increased the size of cellular lipid droplets (Fig. 1C), we isolated TAG from CHO-K1 cells treated under similar experimental conditions (24 h-incubation in standard medium, without added exogenous AA) and analyzed the fatty acid composition. TAG from GPAT2 cells contained ∼6% AA, whereas no AA was detected in TAG from control cells (Fig. 4B). This increase in AA occurred with a concomitant decrease in palmitic acid. The AA composition of the phospholipid fraction did not differ in GPAT2-overexpressing and control cells (results not shown).

### GPAT2 is expressed in mouse and rat spermatic germ cells

**Figure 5:**
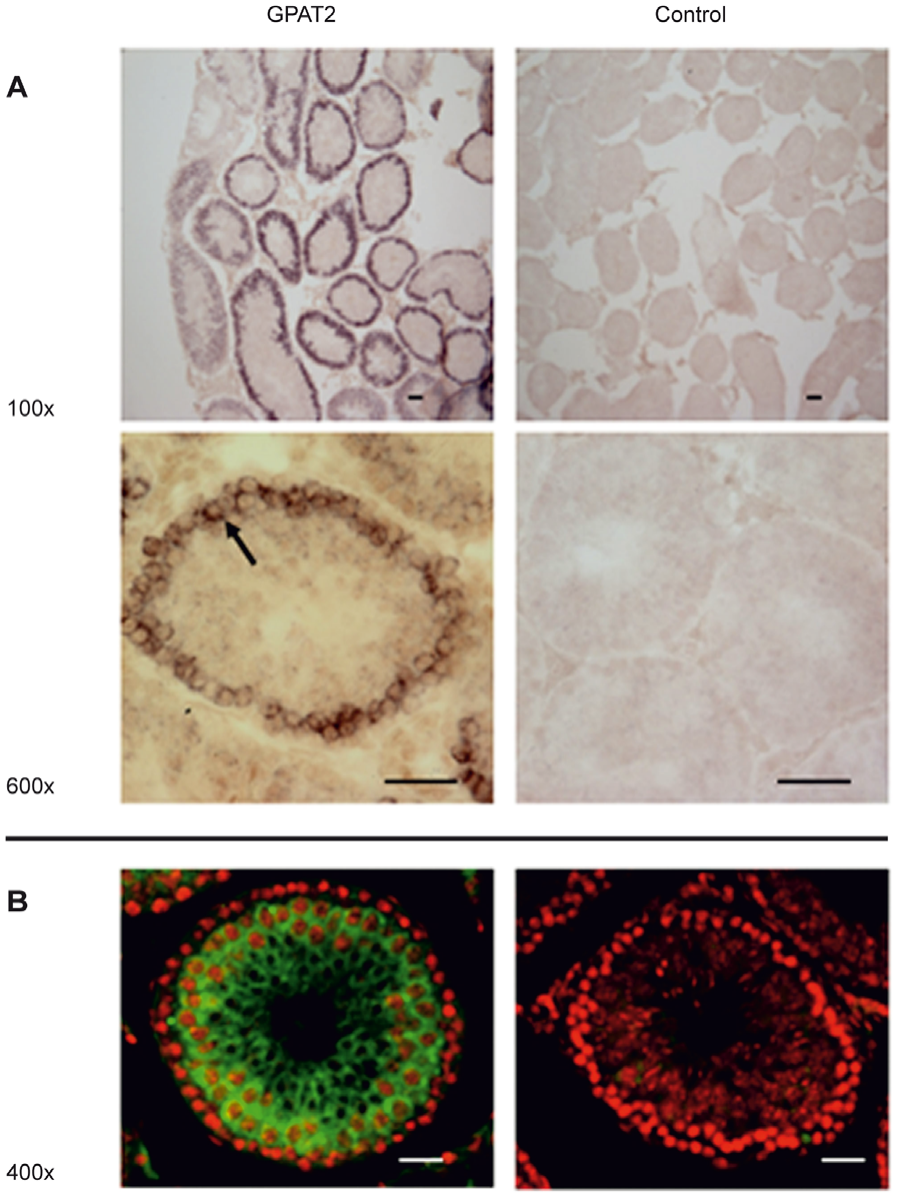
*Gpat2* was expressed in mouse primary spermatocytes. A) Adult mouse testis sections were hybridized in situ with a *Gpat2* specific antisense probe (left panels) and the corresponding sense probe (right panels). Magnification: 100× (first row) and 600× (second row). A strong signal was detected in primary spermatocytes (black arrow). B) GPAT2 protein was detected in adult rat testis by immunofluorescence in the presence (left panel) or absence (right panel) of a specific GPAT2 antibody (green signal). The GPAT2 signal was detected in spermatocytes as well as in spermatides. Nuclei were stained with propidium iodide (red signal). Magnification: 400×. The highest GPAT2 expression was detected in the spermatocytes. Bar: 50 µm.

The expression of *Gpat2* mRNA is 50-fold higher in rat testis than in other tissues [5]. To identify the testicular cell type that expresses *Gpat2*, we performed in situ mRNA hybridization using an anti-sense probe on adult mouse testis sections (Fig. 5A) and immunofluorescence on adult rat testis sections (Fig. 5B). In contrast to *Gpat4* mRNA, which is expressed in both spermatocytes and round spermatides [18], in situ hybridization showed that *Gpat2* mRNA was unequivocally detected only in primary spermatocytes. However, immunofluorescence showed that GPAT2 protein is also expressed in cells undergoing further meiotic and differentiation phases. Neither *Gpat2* mRNA nor protein was expressed in the spermatogonia. Thus, even though transcription of the gene occurs after the first meiotic division, the protein is stable, and is expressed in all of the germ cell types.

### During rat sexual maturation, GPAT2 expression, TAG content and AA in the TAG fraction were maximal at 30 days of age

**Figure 6:**
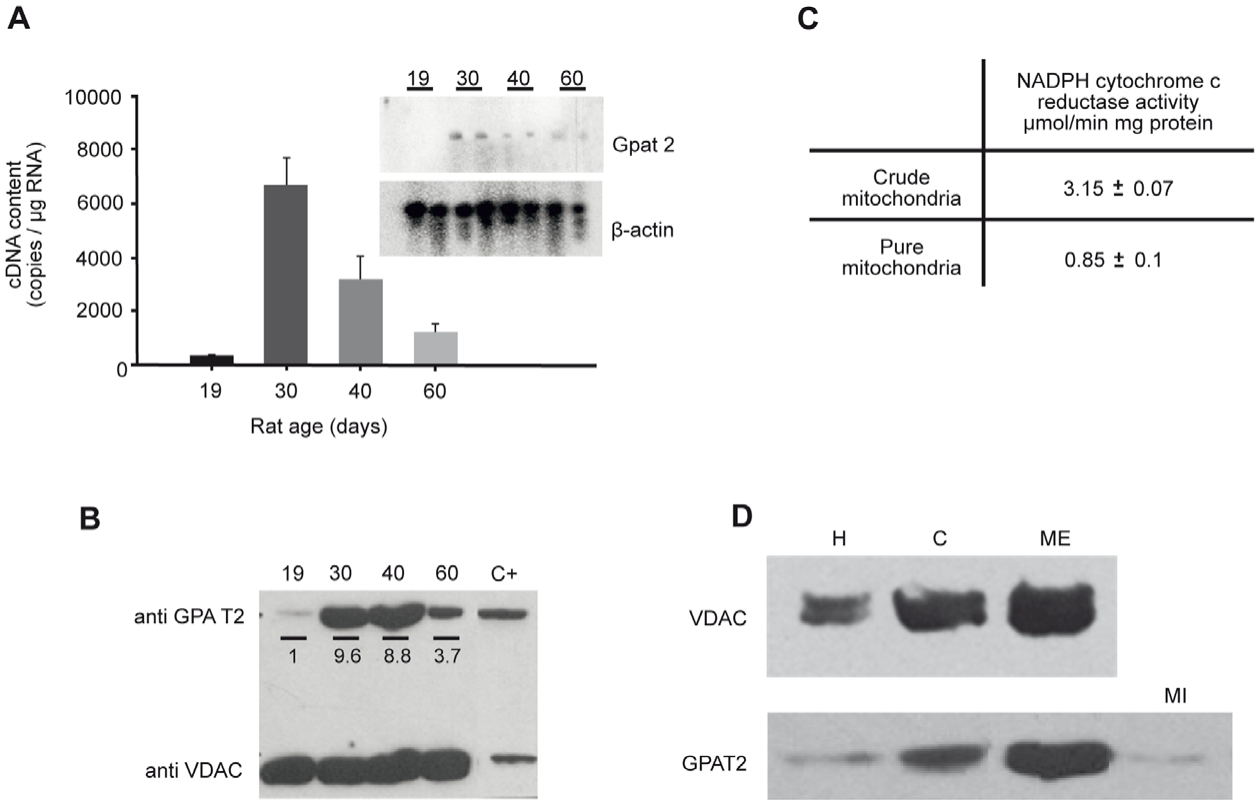
*Gpat2* mRNA and protein expression were maximal in testes from 30-d old rats. Testes from 19, 30, 40 and 60-d old rats were dissected, and either fixed and mounted on glass slides or total mRNA and mitochondria were isolated. A) *Gpat2* mRNA was quantified by real time PCR and validated by Northern blot (insert). Values represent the mean ± SD of three independent experiments. B) Protein expression was measured by western blot with anti-GPAT2 antibody in mitochondria-enriched fraction obtained from 19-d (19), 30-d (30), 40-d (40) and 60-d (60) old rats, as well as in total particulate preparations of GPAT2-overexpressing CHO-K1 cells as a positive control (C+). Anti-voltage-dependent anion channel (VDAC) antibody was used as a loading control. The GPAT2 band corresponded to 80 kDa and VDAC to 30 kDa. Band intensities were quantified using the ImageJ program, and the number below each band represents the intensity ratio of GPAT2/VDAC relative to 19-d old (arbitrarily assigned a value of 1). C) Mitochondrial enrichment was monitored by assaying NADPH-cytochrome c reductase activity and D) by Western blot, probing the membrane with an antibody against the outer membrane protein VDAC. The enrichment of GPAT2 in the outer mitochondrial membrane was detected with anti-GPAT2 primary antibody. H, homogenate, C, crude mitochondria, ME, mitochondria enriched fraction, Mi, microsomes.

**Figure 7:**
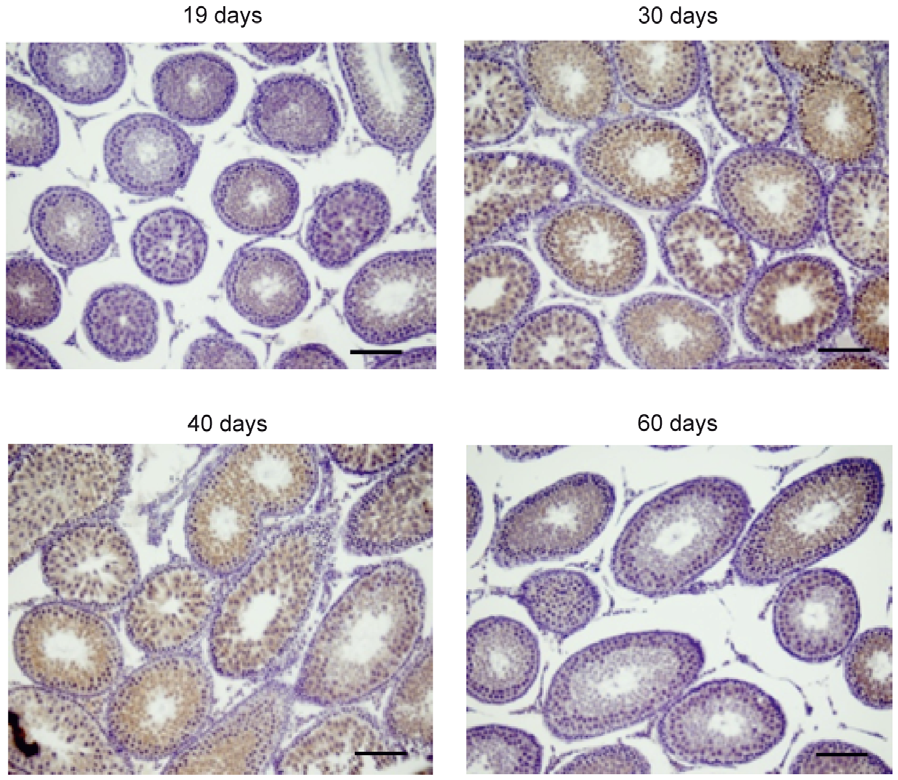
Expression of GPAT2 protein was greatest in 30 d and 40 d rat testis. GPAT2 protein was detected in slides of testes from 19, 30, 40 and 60-d old rats by immunohistochemistry using an anti-GPAT2 antibody (brown signal). Nuclei were counterstained with haematoxylin (blue stain). Magnification: 400×. Bar = 80 µm. The results are representative of three independent experiments.

**Figure 8:**
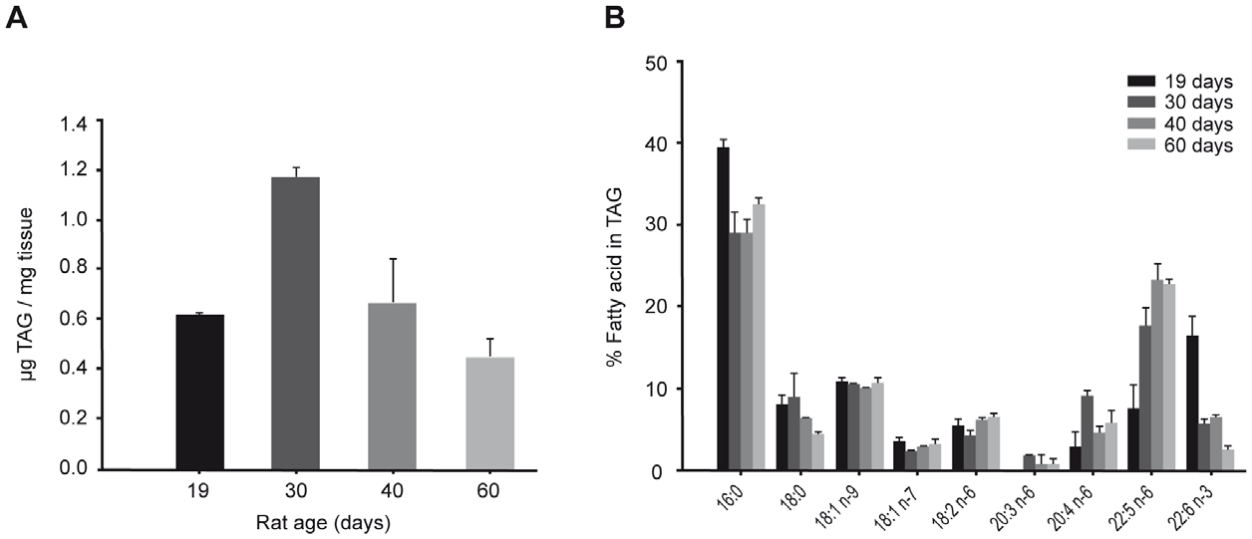
TAG mass and AA content were maximal in testes from 30-d-old rats. Total lipids were isolated from testes from 19, 30, 40 and 60-d old rats. A) Lipids were separated by TLC and charred with 5% sulfuric acid in methanol, and the TAG spot was quantified by image processing. B) Fatty acid composition of the TAG fraction was determined by gas liquid chromatography at different stages of sexual development.

Because GPAT2 was expressed in germ cells, we asked whether its function and expression changed during rat sexual development. We chose four rat developmental stages: infantile (19 d), early (30 d) and late (40 d) juvenile, and postpubertal with mature sperm present in the vas deferens (60 d) [19]. To determine the expression of *Gpat2* during stages of rat sexual development, we examined mRNA abundance by qRT-PCR (Fig. 6A). *Gpat2* mRNA expression was maximal at 30 d of age. This result was validated by Northern blot (Fig. 6A, insert). Protein expression was also evaluated by Western blot in mitochondria-enriched fractions from rat testis at different stages of sexual development (Fig. 6B). Consistent with the mRNA expression, protein expression also peaked at 30 d of age. Mitochondrial purity was validated in fractions from 60 d-old rats by assaying both NADPH cytochrome c reductase activity and the expression of voltage-dependent anion channel protein (VDAC) as mitochondrial markers. Mitochondria-enriched fractions retained about 20% of NADPH cytochrome c reductase activity (Fig. 6C). VDAC western blot (Fig. 6D) confirmed a 1.3 fold mitochondrial enrichment in the purified fraction. We also confirmed the mitochondrial localization of endogenous GPAT2 (Fig. 6D) in subcellular fractions from rat testis. Whereas a strong band was detected in the mitochondrial-enriched fraction, a very weak signal was detected in the microsomal fraction, fainter than the cellular homogenate. To determine whether the increase in GPAT2 protein at 30 d was due to a higher expression in each cell or to an enrichment of spermatocytes that expressed GPAT2, we performed immunohistochemistry with anti-GPAT2 antibody in slides of testes at different ages. To rule out the possibility that variations in the signal were due to differences in the immunohistochemistry procedure; all testes were included in the same paraffin block and processed simultaneously (Fig. 7). The results showed that at 30 d and 40 d GPAT2 was present in a higher percentage of cells and with a stronger signal in each cell. Because in vitro studies showed that GPAT2 overexpression increased AA incorporation into TAG (Fig. 4), we quantified the total testicular TAG content and composition at different stages of sexual development (Fig. 8A). TAG mass was maximal at 30 d of age and the AA esterified in total testicular TAG fraction also peaked at 30 d (Fig. 8B), suggesting that GPAT2 was specifically esterifying AA into TAG during this period.

## Discussion

In the present paper we report a novel feature that distinguishes GPAT2 from other GPAT isoforms: we have identified mitochondrial GPAT2 as an acyltransferase that prefers arachidonoyl-CoA. In order to test the acyl-CoA preference for GPAT2 we performed GPAT assays using different substrates and found that only arachidonoyl-CoA was used. This result was confirmed in two different cell lines. The presence of an LPA product in GPAT assays performed in membranes isolated from cells overexpressing recombinant proteins has been used as a criterion to attribute GPAT activity to these proteins [20], [21]. However, the major product for GPAT1 overexpression was PA, and for GPAT3 and GPAT4 overexpressed in HEK293T and Sf9 cells, only PA increased [22]. We did not detect LPA as a product of the GPAT reaction with overexpressed GPAT2, but we found enhanced PA production when arachidonoyl-CoA was used as substrate, and both GPAT and AGPAT activities were higher in vitro. The activities of both GPAT and AGPAT increased 2-fold with respect to control cells. These findings suggest that the increase in PA may be due to an AGPAT activity for this heterologously-expressed protein, but we cannot exclude the possibility that the protein has dual enzymatic activities. The AGPAT activity detected with oleoyl-CoA also explains why, although we could not detect any GPAT activity with this acyl-CoA, in a previous work we reported an enhancement in TAG synthesis when cells were incubated with exogenous oleate [5]. Although the cDNA-codified GPAT2 protein colocalizes with mitochondria [5], and we confirmed the mitochondrial localization of the endogenous protein in testis (Fig. 6D), endogenous and transfected proteins might differ functionally.

GPAT1, the product of the first mammalian GPAT isoform cloned [23], [24], is also an intrinsic protein of the outer mitochondrial membrane, but it is resistant to NEM inactivation and prefers saturated acyl-CoA substrates like 16∶0- and 18∶0-CoA. GPAT1 activity is highest in mouse and rat liver and adipose tissues. In liver, GPAT1 mRNA abundance is up-regulated by SREBP1c and appears to initiate TAG synthesis when excess carbohydrate or fat calories are consumed [25]. Both GPAT3 and 4 are located in the ER, are inactivated by sulfhydryl reagents like NEM, and use a broad range of acyl-CoA substrates from 12 to 20 carbons [2]. Consistent with their role in TAG synthesis, insulin stimulates the phosphorylation of both GPAT3 and 4 [22]. *Gpat3* mRNA is highly expressed in epididymal adipose tissue as well as in the small intestine of mice, and in kidney, testis, heart, skeletal muscle, and thyroid in humans. *Gpat3* mRNA expression increases 60-fold in 3T3-L1 adipocytes during differentiation, and increases 4.5-fold in white adipose tissue from mice treated for 21 days with the PPARγ agonist rosiglitazone [20]. *Gpat4* mRNA is highly expressed in mouse lipogenic tissues, including liver and both brown and white adipose tissues [26], and it is also highly expressed in testis [18], [26]. GPAT3 and GPAT4 were initially named AGPAT8 and AGPAT6, respectively. Because GPAT3, but not GPAT4, has been reported to have AGPAT activity, it has also been called AGPAT10/GPAT3 [27].

As previously reported [5], the fact that GPAT2 is involved in TAG metabolism was confirmed in different experiments. GPAT2 overexpression increased lipid droplet size by 2-fold and, consistent with the preference of GPAT2 for arachidonoyl-CoA, AA comprised approximately 6% of the TAG fatty acid species from GPAT2-overexpressing cells, whereas no AA was detectable in TAG from control cells. Labeling experiments showed that AA was primarily incorporated into the phospholipid fraction in both control and GPAT2 cells, and that GPAT2 overexpression increased the incorporation of [^14^C]AA into TAG by ∼40% and decreased [^14^C]AA content in phosphatidylethanolamine by the same amount. It has been reported that in U937 cells at 1 µM AA in the medium (similar to the concentration used in our labeling experiments), labeled AA is incorporated at short incubation times in equal amounts into both phosphatidylethanolamine and phosphatidylcholine. However, AA is rapidly recycled from phosphatidylcholine to phosphatidylethanolamine, so that at 3 hr (the time in our labeling experiments), AA content in phosphatidylethanolamine was twice as high as the content in phosphatidylcholine [28]. GPAT2 overexpression may be interfering with phosphatidylethanolamine as an AA acceptor in the Lands cycle, instead directing the AA towards TAG synthesis. It has been proposed that AA becomes incorporated into phospholipids by two different pathways [29]. The major pathway under most physiological conditions is a high-affinity pathway that uses the Land remodeling cycle to incorporate AA into phospholipids when AA is present at low concentrations. The second pathway is a low affinity, high capacity one which incorporates AA primarily into TAG via de novo esterification and results in the accumulation of AA in TAG and diarachidonoyl phospholipids [30]. Because the acyltransferases involved in the phospholipid remodeling pathway are active in both control and GPAT2 cells and because the radioactive AA concentration in the media was low in our experimental conditions (4.3 µM), it is likely that AA is primarily incorporated into phospholipids via the Lands pathway. We propose that GPAT2 may be involved in the low- affinity and high- capacity pathway and that its overexpression directs AA to TAG via the de novo synthesis pathway.

It has been suggested that GPAT4 is involved in the germ-cell proliferation in mouse testis [18]. Although the specific function of GPAT2 in spermatogenesis has not been determined yet, we cannot discard the possibility that it is also involved in cell survival. Because high levels of AA induce apoptosis [31]–[33], and because metabolic pathways that diminish the content of unesterified AA can prevent apoptosis [34], AA turnover is implicated in mammalian cell survival and proliferation. In rat testis, apoptosis aids in maintaining an optimal Sertoli-to-germ-cell ratio, a process that is maximal during the first wave of spermatogenesis and peaks in testis from 25-d old rats. A major feature of mammalian GPAT isoforms 1, 3 and 4 is their central role in the synthesis of stored TAG, evidenced by their high expression in lipogenic tissues like liver and adipose depots, and their fatty acyl-CoA substrates [2]. In contrast, *Gpat2* mRNA and protein are highly expressed in testis, where GPAT2 appears to preferentially esterify AA. This feature, together with the finding that the testis TAG content of AA peaked at 30 d of age, in concert with maximal *Gpat2* mRNA and protein, suggest that GPAT2 may not be involved in TAG synthesis for energy storage, but instead, may regulate the availability of AA within spermatogenic cells. Enhanced GPAT2 activity in spermatogenic cells may allow them to sequester AA into TAG, a function that may be related to cell survival.
